# The effect of inflammatory markers on mortality in patients with acute myocardial infarction

**DOI:** 10.1038/s41598-025-98408-y

**Published:** 2025-04-25

**Authors:** Zhenkun Yang, Yuanjie Li, Taipu Guo, Mingjuan Yang, Yang Chen, Yuxia Gao

**Affiliations:** 1 Department of Cardiology, Tianjin Medical University General Hospital, Tianjin, People’s Republic of China; 2 Department of Anesthesiology, Tianjin Research Institute of Anesthesiology, Tianjin Medical University General Hospital, Tianjin, China; 3 Liverpool Centre for Cardiovascular Science at University of Liverpool, Liverpool John Moores University and Liverpool Heart and Chest Hospital, Liverpool, UK; 4 Department of Cardiovascular and Metabolic Medicine, Institute of Life Course and Medical Sciences, University of Liverpool, Liverpool, UK

**Keywords:** MIMIC-IV, Inflammatory indicator, AMI, Blood cell count, In-hospital mortality, Cardiology, Medical research, Risk factors

## Abstract

Consider that inflammatory factors are associated with short-term mortality in patients with acute myocardial infarction (AMI). In this retrospective analysis of 2,784 AMI patients from the Medical Information Mart for Intensive Care-IV database, we evaluated the impact of inflammatory markers on in-hospital mortality and predicted 30-day and 90-day outcomes. Patients were divided into groups based on in-hospital survival (*n* = 2,364) and mortality (*n* = 420). Analysis of initial hospital admission laboratory data, including inflammatory factors, revealed these factors as independent predictors of in-hospital mortality (Q4 of RDW: OR 1.96, NLR: OR 1.63, SII: OR 1.85, and SIRI: OR 2.23, all *P* < 0.05). Cox proportional hazards models confirmed their significance for predicting 30-day (Q4 of NLR: OR 1.83, SII: OR 1.86, and SIRI: OR 2.01, all *P* < 0.05) and 90-day mortality (Q4 of RDW: OR 1.46, NLR: OR 1.69, SII: OR 1.73, and SIRI: OR 1.72, all *P* < 0.05). Increasing levels of inflammatory markers correlated with higher odds and hazard ratios, as illustrated by Restricted Cubic Spline curves. Kaplan-Meier survival analysis showed better survival rates with lower inflammatory marker levels. Receiver operating characteristic curves demonstrated good predictive performance of individual inflammatory factors, with a new composite marker showing the highest predictive ability (AUC = 0.720). This study underscores the association of inflammatory factors with both hospital and short-term mortality in AMI patients.

**Supplementary Information:**

The online version contains supplementary material available at 10.1038/s41598-025-98408-y.

## Introduction

Acute myocardial infarction (AMI), including ST-segment elevation myocardial infarction (STEMI) and non-ST-segment elevation myocardial infarction (NSTEMI), is a critical cardiac emergency associated with high morbidity and mortality^1^. Patients with AMI have a high hospital mortality rate, reported to be about 7–10%^2–4^, so it is necessary to explore the risk factors associated with mortality.

The pathophysiology of AMI involves myocardial necrosis due to prolonged ischemia, triggering an inflammatory response that exacerbates tissue damage and impairs cardiac function^5^. Inflammatory cytokines, such as Interleukin-1 beta and Tumor Necrosis Factor alpha, further contribute to myocardial injury and adverse outcomes^6–8^. In addition, the formation and rupture of coronary atherosclerotic plaque is itself is related to the inflammatory response. During myocardial infarction, persistent or excessive inflammatory responses can promote ventricular remodeling, leading to ventricular dilatation and cardiac function impairment^9^. Therefore, inflammatory reaction is an important pathophysiological link in the occurrence and development of myocardial infarction, and it has an important relationship with the course and prognosis of myocardial infarction.

Inflammatory markers such as the neutrophil-to-lymphocyte ratio (NLR), platelet-to-lymphocyte ratio (PLR), and monocyte-to-lymphocyte ratio (MLR) have been widely studied for their association with cardiovascular outcomes^10^. Red blood cell distribution width (RDW) has also been identified as an independent predictor of in-hospital and long-term mortality in STEMI patients, with elevated RDW significantly increasing cardiovascular disease risk^11,12^. Additionally, the red cell distribution width-to-platelet ratio (RPR) reflects blood rheological status, with elevated RPR indicating increased red blood cell aggregation and viscosity, potentially leading to microcirculation disorders and adverse cardiovascular events^13^. In patients with coronary heart disease in the intensive care unit (ICU), several markers of inflammation were identified as independent predictors of mortality in the ICU^14^. A prospective cohort study conducted in Tangshan, Hebei Province, China, demonstrated the correlation between systemic immune inflammation index (SII), systemic inflammatory response index, and the risk of cardiovascular disease and all-cause mortality. Furthermore, Xiao et al. observed a U-shaped relationship between SII and all-cause mortality in patients with cardiovascular disease (CVD)^15^. With advances in emergency and intensive care technology, hospital mortality from AMI has decreased, but is still at higher levels.

The purpose of this study was to investigate the relationship between inflammatory markers such as RDW, NLR, PLR, MLR, RPR, SII and SIRI and all-cause in-hospital mortality in AMI and its predictive analysis of short-term (30-day, 90-day) mortality.

## Methods

### Data source

The population data utilized in this retrospective study were sourced from the Medical Information Mart for Intensive Care-IV (MIMIC-IV) database (version 2.2). This comprehensive database, developed and maintained by the Massachusetts Institute of Technology Computational Physiology Laboratory, encompasses extensive medical records of patients admitted to the intensive care units of the Beth Israel Deaconess Medical Center. Access to the database and the requisite certifications (ID 57121385) were secured by one of the authors (Zhenkun Yang). This research project complies with the ethical principles set forth in the Declaration of Helsinki and received approval from the Institutional Review Boards of both the Massachusetts Institute of Technology and the Beth Israel Deaconess Medical Center. Given the standardized nature of the database and the anonymity of participant data, additional ethical approval was deemed unnecessary.

### Study population

**Fig. 1:**
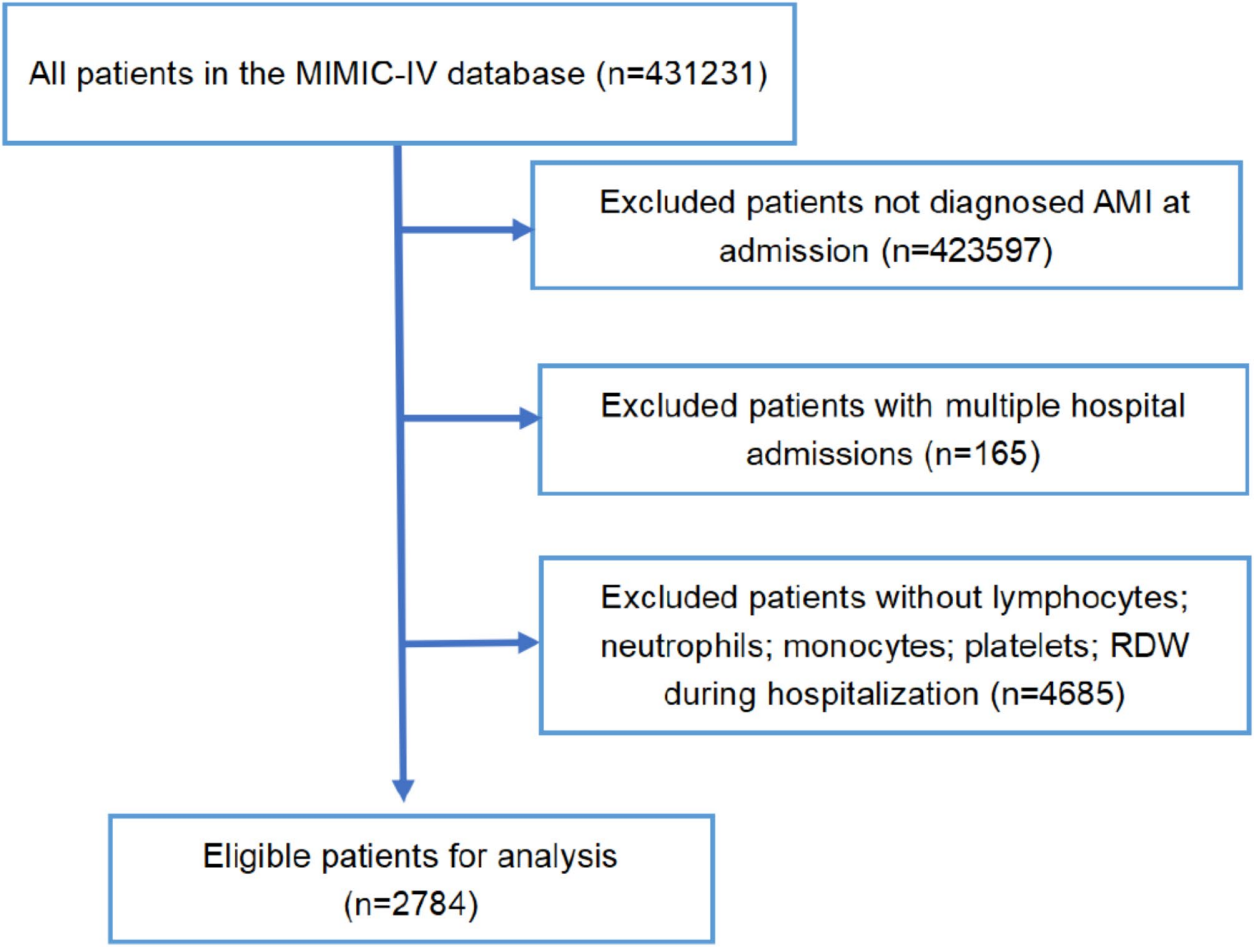
Flow chart illustrating the inclusion and exclusion criteria. AMl, acute myocardial infarction; MlMlC-lV, Medical Information Mart for IntensiveCare-lV, RDW, red blood cell distribution width.

Patients admitted with a diagnosis of AMI, as defined by the International Classification of Diseases (ICD) 9th and 10th editions, were included in the study (Supplementary Table S1). The exclusion criteria comprised the following: (1) age at first admission < 18 years; (2) hospital stay duration < 24 h; (3) patients with duplicate hospitalization records; and (4) incomplete data for neutrophils, lymphocytes, monocytes, platelets, or RDW. Ultimately, 2,784 patients who met the inclusion criteria were enrolled in the statistical analysis, as illustrated in Fig. 1.

### Data extration

Data extraction was performed using Navicat Premium (version 16.1.15) software through the execution of Structured Query Language (SQL). The extracted data encompassed demographic characteristics (age, gender, weight, hight, and race), vital signs (heart rate and blood oxygen saturation), comorbidities (hypertension, type 2 diabetes, atrial fibrillation, congestive heart failure, chronic obstructive pulmonary disease [COPD], cerebrovascular disease, kidney disease, liver disease, peripheral vascular disease, and malignant tumors), laboratory tests (red blood cell counts [RBC], white blood cell counts [WBC], hemoglobin, platelets, blood creatinine, blood urea nitrogen [BUN], sodium, potassium, calcium, glucose, total bilirubin, plasma prothrombin time [PT], and activated partial prothrombin time [APTT]), and disease severity scores at admission (Acute Physiology Score III [APSIII], Oxford Acute Severity Score [OASIS], Sepsis-Related Organ Failure Assessment Score [SOFA], and Systemic Inflammatory Response System [SIRS]). Intervention and surgical procedures, including percutaneous coronary intervention (PCI) and coronary artery bypass grafting (CABG), were also considered (compliance with ICD-10 is detailed in Supplementary Table S1). Pharmacological treatments, such as aspirin, statins, heparin, β-blockers (BBs), angiotensin-converting enzyme inhibitors/angiotensin receptor blockers (ACEI/ARBs), calcium channel blockers (CCBs), and nitrates, were also included. For patients with multiple admissions, the initial admission record was selected, and the corresponding measurement indicators were extracted from the first time point post-admission.

The computational formulas for systemic immune-inflammation index (SII) and systemic inflammation response index (SIRI) were defined as follows: SII = neutrophil count × platelet count / lymphocyte count; SIRI = neutrophil count × monocyte count/lymphocyte count. Body mass index was calculated as weight (kg) divided by height squared (m^2^).

### Mortality outcomes

The primary outcomes of our study were hospital mortality, 30-day mortality, and 90-day mortality in patients with acute myocardial infarction.

### Statistical analysis

For continuous variables, the mean ± standard deviation or the median and interquartile range (IQR) were reported, depending on the data distribution. Group differences were analyzed using one-way analysis of variance or Kruskal-Wallis tests, as appropriate. Differences in categorical variables among groups were assessed using Fisher’s exact test or Chi-squared test, and results were expressed as counts and percentages.

Baseline characteristics of the study cohort were stratified based on the occurrence of in-hospital mortality. To investigate the independent association between inflammatory markers and the primary endpoint, logistic regression analysis was employed to assess factors influencing in-hospital mortality, while 30-day and 90-day mortality were evaluated using Cox proportional hazards models. The cohort was categorized into four quartiles based on the 25th, 50th, and 75th percentiles of inflammatory markers. The first quartile (Q1), representing the lowest values, was designated as the reference group. Adjusted odds ratios (ORs) and hazard ratios (HRs) with 95% confidence intervals (CIs) were calculated for in-hospital, 30-day, and 90-day mortality in the other quartiles relative to Q1. Variables demonstrating statistical significance (*P* < 0.05) in univariate analysis were incorporated as confounders in the multivariable models. Model I: Unadjusted. Model II: Adjusted for age, sex, heart rate, and oxygen saturation. Model III: Model II with additional adjustments for comorbidities, laboratory parameters, clinical severity scores, CABG, and pharmacological treatments. Restricted cubic spline functions with four knots were utilized to examine potential nonlinear relationships between inflammatory markers and the three outcomes. Kaplan-Meier survival curves were constructed to compare the 90-day survival distributions across quartiles of inflammatory markers, and log-rank tests were employed to assess statistical differences. Additionally, the area under the curve (AUC) derived from receiver operating characteristic (ROC) analysis was utilized to evaluate the predictive performance of each inflammatory marker for in-hospital mortality. Simultaneously, the efficacy of combining RDW, NLR, PLR, MLR, RPR, SII, and SIRI to generate a novel predictive marker for in-hospital mortality was assessed.

Furthermore, we conducted a sensitivity analysis to assess the robustness of our findings: (i) Given the significant impact of sepsis and autoimmune diseases on mortality and inflammatory markers, we adjusted for these conditions as confounders to verify the robustness of the primary results; (ii) To avoid potential reverse causality, we excluded patients with a history of malignancy or renal disease and re-evaluated the association between inflammatory markers and mortality outcomes; (iii) Considering the high proportion of patients with comorbid COPD, we excluded these patients from the analysis to further assess the robustness of the results.

All statistical analyses were performed using SPSS (version 27), StataMP-64, and R (version 4.3.2). A two-tailed *P* value < 0.05 was considered statistically significant.

## Results

### Baseline characteristics

A total of 2,784 patients with AMI in the MIMIC-IV database were included in this study. The baseline characteristics are shown in Table 1. Stratified by hospital mortality, 420 patients experienced hospital mortality, while 2,364 survived the hospitalization. The median age of the cohort was 69 years, and 63% were male. Patients who died in the hospital were older, less likely to be male, and had higher risk scores compared to those who survived. They also had a more burden of comorbidities and more abnormal lab results. Levels of studied parameters including RDW, NLR, MLR, RPR, SII and SIRI were significantly higher in the hospital mortality group compared to the survivor group. However, PLR did not show a statistically significant difference between the two groups (*P* = 0.085).

**Table 1:** Baseline characteristics of hospital survival and hospital mortality.

Category	All (*N* = 2784)	Hospital survival (*N* = 2364)	Hospital mortality (*N* = 420)	*P*
Age (year)	69 (60, 78)	69 (59, 78)	73 (64, 81)	< 0.001
Male (n, %)	1754 (63%)	1508 (63.8%)	246 (58.6%)	0.043
Race (n, %)				0.323
White	1760 (63.2%)	1508 (63.8%)	252 (60%)	
Black	214 (7.7%)	180 (7.6%)	34 (8.1%)	
Others	810 (29.1%)	676 (28.6%)	134 (31.9%)	
BMI (kg/m^2^)	27.23 (23.68, 31.60)	27.25 (23.70, 31.64)	27.18 (23.62, 31.52)	0.930
Heart rate (bpm)	100 (78.5, 113)	99 (77.5, 111)	107 (91.25, 123)	< 0.001
Blood oxygen saturation (%)	93 (71, 97)	93 (72, 97)	85 (64, 97)	< 0.001
Comorbidities (n, %)				
Hypertension	996 (35.8%)	878 (37.1%)	118 (28.1%)	< 0.001
Type 2 diabetes	116 (41.8%)	982 (41.5%)	181 (43.1%)	0.555
Congestive heart failure	1453 (52.2%)	1193 (50.5%)	260 (61.9%)	< 0.001
Atrial fibrillation	1022 (36.7%)	826 (34.9%)	196 (46.7%)	< 0.001
COPD	665 (23.9%)	549 (23.2%)	116 (27.6%)	0.054
Peripheral vascular disease	359 (12.9%)	286 (12.1%)	73 (17.4%)	0.004
Cerebrovascular disease	416 (14.9%)	324 (13.7%)	92 (21.9%)	< 0.001
Hepatic disease	221 (7.9%)	150 (6.3%)	71 (16.9%)	< 0.001
Renal disease	890 (32%)	731 (30.9%)	159 (37.9%)	0.005
Malignant cancer	275 (9.9%)	219 (9.3%)	56 (13.3%)	0.013
Laboratory tests				
WBC (K/uL)	10.3 (7.5, 14.4)	9.90 (7.4, 13.6)	13.4 (9, 19.4)	< 0.001
RBC (m/uL)	3.83 (3.20, 4.41)	3.88 (3.24, 4.56)	3.58 (2.97, 4.14)	< 0.001
Platelet (K/uL)	199 (153, 257)	200 (158, 256)	189 (137, 266.5)	0.014
Hemoglobin (g/dL)	11.4 (9.4, 13.2)	11.5 (9.6, 13.3)	10.6 (9, 12.3)	< 0.001
Creatinine (mg/dL)	1.2 (0.90, 1.7)	1.1 (0.8, 1.6)	1.5 (1.1, 2.2)	< 0.001
BUN (mg/dL)	23(16, 39)	22 (15, 36)	32 (20, 50)	< 0.001
Sodium (mg/dL)	139 (136, 141)	139 (136, 141)	138 (135, 141)	0.019
Calcium (mg/dL)	8.6 (8.1, 9.1)	8.70 (8.20, 9.1)	8.30 (7.70, 8.8)	< 0.001
Potassium (mg/dL)	4.20 (3.9, 4.60)	4.20 (3.9, 4.6)	4.30 (3.8, 4.9)	0.008
Glucose (mg/dL)	135 (107, 191)	132 (106, 184)	161 (117.25, 232.5)	< 0.001
Total bilirubin (mg/dL)	0.6 (0.4, 0.9)	0.6 (0.4, 0.8)	0.70 (0.4, 1.2)	< 0.001
PT (s)	13.1 (11.9, 15.5)	12.9 (11.8, 14.9)	14.8 (13.1, 19.30)	< 0.001
APTT (s)	34.60 (28.5, 55.48)	34.60 (28.5, 53.9)	34.80 (28.63, 66.48)	0.061
OASIS	33 (26, 40)	32 (26, 38)	42 (35, 49)	< 0.001
SIRS	3 (2, 3)	3 (2, 3)	3 (2, 4)	< 0.001
SOFA	5 (3, 9)	5 (3, 8)	10 (7, 13)	< 0.001
APSIII	46 (32, 67)	42 (30, 59.75)	77 (60, 101)	< 0.001
Intervention (%)				
PCI	251 (9%)	223 (9.4%)	28 (6.7%)	0.078
CABG	565 (20.3%)	552 (23.4%)	13 (3.1%)	< 0.001
Drugs (%)				
Aspirin	2309 (82.9%)	2000 (84.6%)	309 (73.6%)	< 0.001
Statins	2260 (81.2%)	1983 (83.9%)	277 (66%)	< 0.001
Heparin	2603 (93.5%)	2206 (93.3%)	397 (94.5%)	0.392
BBs	2304 (82.8%)	2069 (87.5%)	235 (56%)	< 0.001
ACEI/ARBs	1205 (43.3%)	1139 (48.2%)	66 (15.7%)	< 0.001
CCBs	456 (16.4%)	404 (17.1%)	52 (12.4%)	0.015
Nitrates	1340 (48.1%)	1219 (51.6%)	121 (28.8%)	< 0.001
Inflammatory indicators				
RDW	14.2 (13.2, 15.8)	14 (13.2, 15.5)	15.2 (14, 17.2)	< 0.001
NLR	6.59 (3.68, 11.98)	6.05 (3.53, 10.96)	10.20 (5.73, 20.34)	< 0.001
PLR	117.80 (53.32, 213.18)	116.98 (56.67, 204.50)	131.92 (19.59, 275.57)	0.085
MLR	0.50 (0.29, 0.92)	0.47 (0.27, 0.86)	0.76 (0.43, 1.29)	< 0.001
RPR	0.07 (0.06, 0.10)	0.07 (0.05, 0.09)	0.08 (0.07, 0.13)	< 0.001
SII	1295.12 (677.51, 2568.30)	1222.26 (655.60, 2327.80)	2091.40 (885.07, 3979.55)	< 0.001
SIRI	6.62 (2.41, 28.73)	5.64 (2.20, 22.59)	15.11 (5.79, 58.94)	< 0.001

BMI, body mass index; COPD, chronic obstructive pulmonary disease; WBC, white blood cell counts; RBC, red blood cell counts; BUN, blood Urea nitrogen; PT, plasma prothrombin time; APTT, activated partial prothrombin time; OASIS, Oxford acute severity score; APSIII, acute physiology score III; SOFA, sepsis-related organ failure assessment score; SIRS, systemic inflammatory response score system; PCI, percutaneous coronary intervention; CABG, coronary artery bypass grafting; BBs, β-blockers; ACEI/ARBs, angiotensin converting enzyme inhibitors/angiotensin receptor blockers; CCBs, calcium channel blockers, RDW, red blood cell distribution width; NLR, neutrophil to lymphocyte ratio; PLR, platelet to lymphocyte ratio; MLR, monocyte to lymphocyte ratio; RPR, red blood cell volume distribution width to platelet ratio; SII, systemic immune inflammation index; SIRI, systemic inflammatory response index.

### Effect of inflammatory markers on in-hospital mortality in AMI

The inflammatory factors were then divided into four groups according to the quartile: RDW: Q1:11.9–13.2; Q2: 13.2–14.2; Q3: 14.2–15.8; Q4: 15.8–23.3; NLR: Q1: 0.51–3.69; Q2: 3.69–6.59; Q3: 6.59-12.00; Q4: 12.00-79.50; PLR: Q1: 0.53–53.39; Q2: 53.39–117.80; Q3: 117.80-213.17; Q4: 213.17-1166.67; MLR: Q1:0.05–0.29; Q2:0.29–0.50;Q3: 0.50–0.92; Q4: 0.92–4.59; RPR: Q1: 0.03–0.06; Q2: 0.06–0.07; Q3: 0.07–0.10; Q4: 0.10–0.55; SII: Q1:41.00-766.53; Q2: 766.53-1295.12; Q3: 1295.12-2568.27; Q4: 2568.27-17736.67; SIRI: Q1: 0.19-0.2.41; Q2: 2.41–6.62; Q3: 6.62–28.69; Q4: 28.69-1837.44.

In a multivariate Logistic regression model adjusted for confounders (Model III), continuous RDW (OR 1.07, *P* = 0.025), NLR (OR 1.01, *P* = 0.024), RPR (OR 9.02, *P* = 0.002), and SII (OR 1.04, *P* = 0.042) were risk factors for in-hospital mortality. Relative to the Q1, the Q4 of RDW (OR = 1.96, 95% CI: 1.21–3.17, *P* = 0.006), NLR (OR = 1.63, 95% CI: 1.08–2.47, *P* = 0.019), SII (OR = 1.85, 95% CI: 1.21–2.85, *P* = 0.005), and SIRI (OR = 2.23, 95% CI: 1.42–3.50, *P* < 0.001) were identified as independent risk factors for in-hospital mortality among AMI patients. Furthermore, as RDW and SIRI values increased, the risk of in-hospital mortality also escalated. Trends for PLR were statistically significant (*P* = 0.028). However, no significant association was found between MLR, RPR, and in-hospital mortality (Table 2).

**Table 2:** The association of each inflammatory indicator with in-hospital mortality.

Category		Model I			Model II			Model III		
		OR (95% CI)	*P*	*P* for trend	OR (95% CI)	*P*	*P* for trend	OR (95% CI)	*P*	*P* for trend
Continuous RDW		1.18 (1.14, 1.23)	< 0.001		1.15 (1.11, 1.20)	< 0.001		1.07 (1.01, 1.13)	0.025	
Quartiles of RDW	Q1 (*N* = 734)	*Ref*		< 0.001	*Ref*		< 0.001	*Ref*		0.003
	Q2 (*N* = 687)	1.73 (1.19, 2.51)	0.004		1.52 (1.04, 2.21)	0.030		0.98 (0.62, 1.56)	0.930	
	Q3 (*N* = 662)	2.88 (2.03, 4.08)	< 0.001		2.38 (1.66, 3.41)	< 0.001		1.58 (1.01, 2.48)	0.047	
	Q4 (*N* = 701)	4.66 (3.34, 6.50)	< 0.001		3.74 (2.65, 5.28)	< 0.001		1.96 (1.21, 3.17)	0.006	
Continuous NLR		1.03 (1.02, 1.04)	< 0.001		1.03 (1.02, 1.03)	< 0.001		1.01 (1.00, 1.02)	0.024	
Quartiles of NLR	Q1 (*N* = 696)	*Ref*		< 0.001	*Ref*		< 0.001	*Ref*		0.094
	Q2 (*N* = 696)	1.32 (0.92, 1.92)	0.136		1.31 (0.91, 1.91)	0.151		1.19 (0.76, 1.86)	0.440	
	Q3 (*N* = 696)	2.36 (1.68, 3.31)	< 0.001		2.14 (1.52, 3.02)	< 0.001		1.46 (0.97, 2.21)	0.073	
	Q4 (*N* = 696)	3.98 (2.88, 5.50)	< 0.001		3.51 (2.53, 4.87)	< 0.001		1.63 (1.08, 2.47)	0.019	
Continuous PLR		1.01 (1.00, 1.02)	< 0.001		1.00 (1.00, 1.01)	< 0.001		1.00 (1.00, 1.00)	0.462	
Quartiles of PLR	Q1 (*N* = 696)	*Ref*		< 0.001	*Ref*		< 0.001	*Ref*		0.028
	Q2 (*N* = 696)	1.18 (0.84, 1.65)	0.343		1.12 (0.79, 1.57)	0.532		1.07 (0.70, 1.62)	0.761	
	Q3 (*N* = 696)	2.02 (1.47, 2.76)	< 0.001		2.00 (1.46, 2.75)	< 0.001		1.70 (1.13, 2.55)	0.010	
	Q4 (*N* = 696)	2.27 (1.67, 3.09)	< 0.001		1.99 (1.45, 2.72)	< 0.001		1.42 (0.95, 2.13)	0.086	
Continuous MLR		1.47 (1.32, 1.62)	< 0.001		1.40 (1.27, 1.55)	< 0.001		1.11 (0.98, 1.24)	0.084	
Quartiles of MLR	Q1 (*N* = 698)	*Ref*		< 0.001	*Ref*		< 0.001	*Ref*		0.103
	Q2 (*N* = 694)	1.27 (0.89, 1.82)	0.192		1.18 (0.83, 1.70)	0.358		1.12 (0.71, 1.76)	0.624	
	Q3 (*N* = 696)	2.15 (1.54, 2.99)	< 0.001		1.85 (1.32, 2.59)	< 0.001		1.56 (1.02, 2.38)	0.040	
	Q4 (*N* = 696)	3.41 (2.49, 4.68)	< 0.001		2.87 (2.07, 3.96)	< 0.001		1.48 (0.98, 2.24)	0.052	
Continuous RPR		19.24 (7.65, 48.37)	< 0.001		14.44 (5.63, 27.04)	< 0.001		9.02 (2.21, 16.90)	0.002	
Quartiles of RPR	Q1 (*N* = 698)	*Ref*		< 0.001	*Ref*		< 0.001	*Ref*		0.348
	Q2 (*N* = 694)	1.13 (0.83, 1.55)	0.444		1.16 (0.84, 1.60)	0.365		1.15 (0.72, 1.84)	0.564	
	Q3 (*N* = 696)	1.00 (0.72, 1.38)	0.984		0.96 (0.69, 1.33)	0.808		0.74 (0.48, 1.12)	0.156	
	Q4 (*N* = 696)	2.22 (1.66, 2.96)	< 0.001		1.94 (1.44, 2.60)	< 0.001		0.89 (0.54, 1.45)	0.631	
Continuous SII		1.00 (1.00, 1.01)	< 0.001		1.00 (1.00, 1.01)	< 0.001		1.04 (1.01, 1.11)	0.042	
Quartiles of SII	Q1 (*N* = 696)	*Ref*		< 0.001	*Ref*		< 0.001	*Ref*		0.007
	Q2 (*N* = 696)	0.93 (0.65, 1.31)	0.659		0.91 (0.64, 1.30)	0.614		1.01 (0.66, 1.55)	0.955	
	Q3 (*N* = 696)	1.49 (1.09, 2.05)	0.013		1.43 (1.04, 1.97)	0.029		1.14 (0.76, 1.71)	0.541	
	Q4 (*N* = 696)	2.76 (2.05, 3.71)	< 0.001		2.51 (1.86, 3.39)	< 0.001		1.85 (1.21, 2.85)	0.005	
Continous SIRI		1.00 (1.00, 1.01)	< 0.001		1.00 (1.00, 1.01)	< 0.001		1.00 (0.89, 1.03)	0.290	
Quartiles of SIRI	Q1 (*N* = 696)	*Ref*		< 0.001	*Ref*		< 0.001	*Ref*		< 0.001
	Q2 (*N* = 696)	1.41 (0.97, 2.06)	0.072		1.28 (0.88, 1.88)	0.198		1.10 (0.70, 1.73)	0.680	
	Q3 (*N* = 696)	3.24 (2.31, 4.55)	< 0.001		2.79 (1.98, 3.94)	< 0.001		1.79 (1.17, 2.74)	0.007	
	Q4 (*N* = 696)	3.68 (2.63, 5.16)	< 0.001		3.53 (2.51, 4.95)	< 0.001		2.23 (1.42, 3.50)	< 0.001	

Mode I: unadjusted; model II: adjusted by age, sex, heart rate, and oxygen saturation; model III: model II further adjusted by hypertension, atrial fibrillation, congestive heart failure, cerebrovascular disease, kidney disease, liver disease, peripheral vascular disease, malignant tumor, red blood cell counts, white blood cell counts, hemoglobin, platelet counts, creatinine, blood Urea nitrogen, electrolytes, glucose, total bilirubin, plasma prothrombin time, acute physiology score III, Oxford acute severity score, Sepsis-Related organ failure assessment score, systemic inflammatory response score system, coronary artery bypass grafting, aspirin, Statins, β-blockers, angiotensin converting enzyme inhibitors/angiotensin receptor blockers, calcium channel blockers, nitrates. OR, odds ratio; CI, confidence interval; Q, quartile; ref, reference; RDW, red blood cell distribution width; NLR, neutrophil to lymphocyte ratio; PLR, platelet to lymphocyte ratio; MLR, monocyte to lymphocyte ratio; RPR, red blood cell volume distribution width to platelet ratio; SII, systemic immune inflammation index; SIRI, systemic inflammatory response index.

**Fig. 2:**
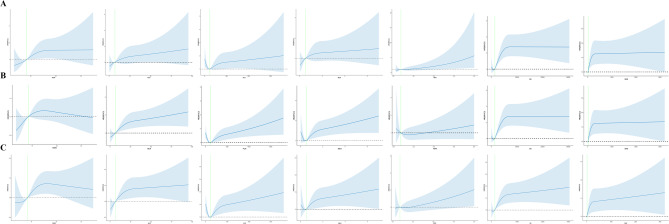
Restricted cubic spline function between inflammation indicators and in-hospital mortality (**A**). Restricted cubic spline function between inflammation indicators and hospital 30-day mortality (**B**). Restricted cubic spline function between inflammation indicators and hospital 90-day mortality (**C**). RDW, red blood cell distribution width; NLR, neutrophil to lymphocyte ratio; PLR, platelet to lymphocyte ratio; MLR, monocyte to lymphocyte ratio; RPR, red blood cell volume distribution width to platelet ratio; SII, systemic immune inflammation index; SIRI, systemic inflammatory response index; OR, odds ratio; CI, confidence interval; HR, hazard ratio.

RCS visualized the relationship between inflammatory markers and in-hospital mortality. In Fig. 2A, the in-hospital mortality of patients with AMI exhibited a non-linear relationship with inflammatory markers (*P* < 0.001). PLR and SII showed U-shaped associations, with the lowest risk at PLR = 117.92 and SII = 1389.61, followed by a gradual increase that plateaued.

### Effect of inflammatory markers on 30-day mortality in AMI

In multivariate Cox proportional hazards models, adjusted for confounders (Model III), Continuous NLR (HR 1.01, *P* < 0.001), SII (HR 1.00, *P* < 0.001), and SIRI (HR 1.00, *P* = 0.021) were risk factors for 30-day mortality. Relative to the Q1, the Q4 of NLR (OR = 1.83, 95% CI: 1.36–2.46, *P* < 0.001), SII (OR = 1.86, 95% CI: 1.39–2.50, *P* < 0.001), and SIRI (OR = 2.01, 95% CI: 1.47–2.76, *P* < 0.001) were identified as independent risk factors for 30-day mortality among AMI patients. Furthermore, as the values of NLR and SIRI increased, the risk of 30-day mortality also escalated. The trends for PLR were statistically significant (*P* = 0.039). However, no significant association was observed between RDW, MLR, RPR, and 30-day mortality (Table 3).

**Table 3:** The association of each inflammatory indicator with hospital 30-day mortality.

Category		Model I			Model II			Model III		
		OR (95% CI)	*P*	*P* for trend	OR (95% CI)	*P*	*P* for trend	OR (95% CI)	*P*	*P* for trend
Continuous RDW		1.14 (1.11, 1.17)	< 0.001		1.11 (1.08, 1.14)	< 0.001		1.03 (0.98, 1.07)	0.176	
Quartiles of RDW	Q1 (*N* = 734)	*Ref*		< 0.001	*Ref*		< 0.001	*Ref*		0.052
	Q2 (*N* = 687)	1.77 (1.30, 2.41)	< 0.001		1.54 (1.13, 2.11)	0.006		0.94 (0.68, 1.30)	0.706	
	Q3 (*N* = 662)	2.55 (1.90, 3.42)	< 0.001		2.08 (1.54, 2.80)	< 0.001		1.17 (0.85, 1.60)	0.339	
	Q4 (*N* = 701)	4.13 (3.13, 5.44)	< 0.001		3.27 (2.46, 4.34)	< 0.001		1.36 (0.97, 1.90)	0.071	
Continuous NLR		1.02 (1.02, 1.03)	< 0.001		1.02 (1.02, 1.03)	< 0.001		1.01 (1.00, 1.01)	< 0.001	
Quartiles of NLR	Q1 (*N* = 696)	*Ref*		< 0.001	*Ref*		< 0.001	*Ref*		< 0.001
	Q2 (*N* = 696)	1.28 (0.93, 1.77)	0.133		1.27 (0.92, 1.75)	0.150		1.14 (0.82, 1.59)	0.428	
	Q3 (*N* = 696)	2.47 (1.85, 3.29)	< 0.001		2.27 (1.70, 3.04)	< 0.001		1.53 (1.13, 2.07)	0.006	
	Q4 (*N* = 696)	4.06 (3.09, 5.35)	< 0.001		3.60 (2.73, 4.74)	< 0.001		1.83 (1.36, 2.46)	< 0.001	
Continuous PLR		1.01 (1.00, 1.01)	< 0.001		1.00 (1.00, 1.01)	< 0.001		1.00 (1.00, 1.01)	0.085	
Quartiles of PLR	Q1 (*N* = 696)	*Ref*		< 0.001	*Ref*		< 0.001	*Ref*		0.039
	Q2 (*N* = 696)	1.17 (0.88, 1.55)	0.285		1.10 (0.83, 1.46)	< 0.512		0.96 (0.72, 1.29)	0.801	
	Q3 (*N* = 696)	1.85 (1.42, 2.41)	< 0.001		1.81 (1.39, 2.36)	< 0.001		1.33 (1.00, 1.75)	0.049	
	Q4 (*N* = 696)	2.39 (1.86, 3.07)	< 0.001		2.07 (1.60, 2.66)	< 0.001		1.26 (0.96, 1.65)	0.103	
Continuous MLR		1.31 (1.24, 1.37)	< 0.001		1.27 (1.21, 1.34)	< 0.001		1.10 (1.03, 1.18)	0.004	
Quartiles of MLR	Q1 (*N* = 698)	*Ref*		< 0.001	*Ref*		< 0.001	*Ref*		0.065
	Q2 (*N* = 694)	1.12 (0.83, 1.51)	0.461		1.05 (0.78, 1.42)	0.750		0.85 (0.62, 1.16)	0.307	
	Q3 (*N* = 696)	1.77 (1.35, 2.33)	< 0.001		1.52 (1.16, 2.01)	0.003		1.08 (0.81, 1.45)	0.596	
	Q4 (*N* = 696)	3.04 (2.36, 3.91)	< 0.001		2.52 (1.95, 3.27)	< 0.001		1.21 (0.91, 1.59)	0.189	
Continuous RPR		5.70 (3.28, 9.91)	< 0.001		4.17 (2.35, 7.40)	< 0.001		1.75 (0.73, 4.16)	0.208	
Quartiles of RPR	Q1 (*N* = 698)	*Ref*		< 0.001	*Ref*		< 0.001	*Ref*		0.144
	Q2 (*N* = 694)	1.22 (0.94, 1.59)	0.130		1.28 (0.98, 1.66)	0.068		1.23 (0.89, 1.69)	0.207	
	Q3 (*N* = 696)	1.08 (0.83, 1.41)	0.572		1.05 (0.80, 1.37)	0.732		0.80 (0.60, 1.07)	0.126	
	Q4 (*N* = 696)	2.01 (1.59, 2.56)	< 0.001		1.75 (1.37, 2.22)	< 0.001		0.77 (0.55, 1.08)	0.124	
Continuous SII		1.00 (1.00, 1.00)	< 0.001		1.00 (1.00, 1.00)	< 0.001		1.00 (1.00, 1.00)	< 0.001	
Quartiles of SII	Q1 (*N* = 696)	*Ref*		< 0.001	*Ref*		< 0.001	*Ref*		< 0.001
	Q2 (*N* = 696)	0.94 (0.70, 1.26)	0.681		0.93 (0.69, 1.25)	0.608		1.09 (0.80, 1.48)	0.583	
	Q3 (*N* = 696)	1.53 (1.17, 2.00)	0.002		1.46 (1.12, 1.91)	0.006		1.20 (0.90, 1.60)	0.214	
	Q4 (*N* = 696)	2.86 (2.24, 3.65)	< 0.001		2.58 (2.02, 3.30)	< 0.001		1.86 (1.39, 2.50)	< 0.001	
Continous SIRI		1.00 (1.00, 1.01)	< 0.001		1.00 (1.00, 1.01)	< 0.001		1.00 (1.00, 1.00)	0.021	
Quartiles of SIRI	Q1 (*N* = 696)	*Ref*		< 0.001	*Ref*		< 0.001	*Ref*		< 0.001
	Q2 (*N* = 696)	1.32 (0.96, 1.81)	0.087		1.21 (0.88, 1.66)	0.235		1.03 (0.74, 1.42)	0.864	
	Q3 (*N* = 696)	3.00 (2.27, 3.96)	< 0.001		2.57 (1.94, 3.40)	< 0.001		1.64 (1.21, 2.22)	0.001	
	Q4 (*N* = 696)	3.21 (2.43, 4.23)	< 0.001		3.03 (2.30, 4.00)	< 0.001		2.01 (1.47, 2.76)	< 0.001	

Mode I: unadjusted; model II: adjusted by age, sex, heart rate, and oxygen saturation; model III: model II further adjusted by hypertension, atrial fibrillation, congestive heart failure, cerebrovascular disease, kidney disease, liver disease, peripheral vascular disease, malignant tumor, red blood cell counts, white blood cell counts, hemoglobin, platelet counts, creatinine, blood Urea nitrogen, electrolytes, glucose, total bilirubin, plasma prothrombin time, acute physiology score III, Oxford acute severity score, Sepsis-Related organ failure assessment score, systemic inflammatory response score system, coronary artery bypass grafting, aspirin, Statins, β-blockers, angiotensin converting enzyme inhibitors/angiotensin receptor blockers, calcium channel blockers, nitrates. HR, hazard ratio; CI, confidence interval; Q, quartile; ref, reference; RDW, red blood cell distribution width; NLR, neutrophil to lymphocyte ratio; PLR, platelet to lymphocyte ratio; MLR, monocyte to lymphocyte ratio; RPR, red blood cell volume distribution width to platelet ratio; SII, systemic immune inflammation index; SIRI, systemic inflammatory response index.

**Fig. 3:**
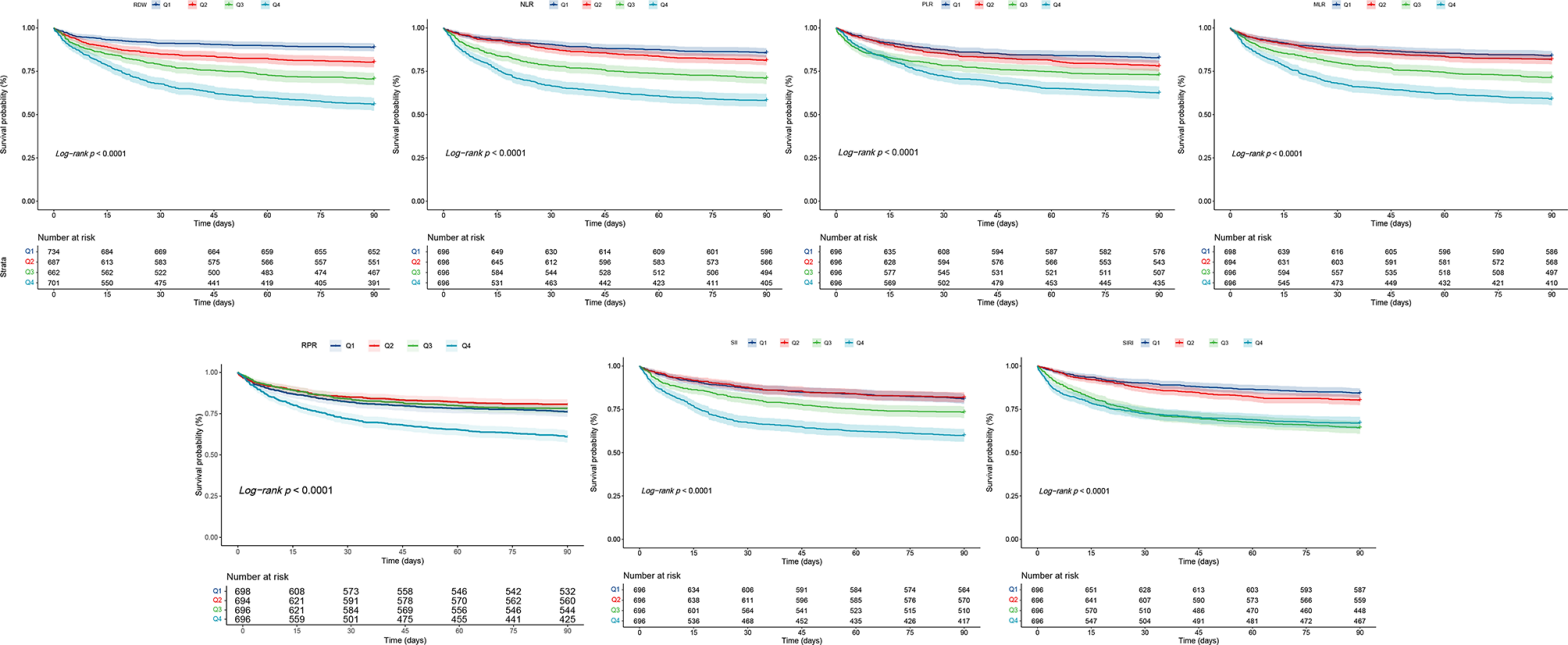
Kaplan-Mill survival analysis curves of all-cause mortality in patients with hospital 90-day mortality. RDW, red blood cell distribution width; NLR, neutrophil to lymphocyte ratio; PLR, platelet to lymphocyte ratio; MLR, monocyte to lymphocyte ratio; RPR, red blood cell volume distribution width to platelet ratio; SII, systemic immune inflammation index; SIRI, systemic inflammatory response index.

RCS curves showed a nonlinear relationship between 30-day mortality and inflammatory markers in AMI patients (*P* < 0.001). The risk of death increased with elevated inflammatory markers (Fig. 2B). Kaplan-Meier survival curves were generated based on quartiles of the inflammatory markers (Fig. 3). The curves illustrate a decrease in the 30-day survival probability of patients as the quartiles of inflammatory markers increased (*P* < 0.001).

### Effect of inflammatory markers on 90-day mortality in AMI

In a multivariate analysis, continuous RDW (HR 1.04, *P* = 0.028), NLR (HR 1.01, *P* < 0.001), MLR (HR 1.08, *P* = 0.015), RPR (HR 2.15, *P* = 0.034), SII (HR 1.00, *P* = 0.002), SIRI (HR 1.00, *P* = 0.019) were risk factors for 90-day mortality. Relative to the Q1, the Q4 of RDW (OR = 1.46, 95% CI: 1.09–1.96, *P* = 0.010), NLR (OR = 1.69, 95% CI: 1.32–2.17, *P* < 0.001), SII (OR = 1.73, 95% CI: 1.34–2.22, *P* < 0.001), and SIRI (OR = 1.72, 95% CI: 1.32–2.23, *P* < 0.001) were identified as independent risk factors for 90-day mortality among AMI patients. Furthermore, as NLR and SIRI values increased, the risk of 90-day mortality also escalated. However, no significant association was observed between PLR, MLR, RPR, and 90-day mortality (Table 4).

**Table 4:** The association of each inflammatory indicator with hospital 90-day mortality.

Category		Model I			Model II			Model III		
		OR (95% CI)	*P*	*P* for trend	OR (95% CI)	*P*	*P* for trend	OR (95% CI)	*P*	*P* for trend
Continuous RDW		1.14 (1.11, 1.17)	< 0.001		1.11 (1.08, 1.14)	< 0.001		1.04 (1.01, 1.07)	0.028	
Quartiles of RDW	Q1 (*N* = 734)	*Ref*		< 0.001	*Ref*		< 0.001	*Ref*		0.009
	Q2 (*N* = 687)	1.86 (1.41, 2.45)	< 0.001		1.60 (1.22, 2.11)	< 0.001		1.02 (0.77, 1.35)	0.894	
	Q3 (*N* = 662)	2.92 (2.25, 3.78)	< 0.001		2.33 (1.79, 3.02)	< 0.001		1.31 (0.99, 1.72)	0.058	
	Q4 (*N* = 701)	4.80 (3.77, 6.13)	< 0.001		3.73 (2.91, 4.79)	< 0.001		1.46 (1.09, 1.96)	0.010	
Continuous NLR		1.02 (1.02, 1.03)	< 0.001		1.02 (1.02, 1.03)	< 0.001		1.01 (1.00, 1.01)	< 0.001	
Quartiles of NLR	Q1 (*N* = 696)	*Ref*		< 0.001	*Ref*		< 0.001	*Ref*		< 0.001
	Q2 (*N* = 696)	1.32 (1.02, 1.71)	0.037		1.31 (1.01, 1.71)	0.040		1.22 (0.94, 1.60)	0.142	
	Q3 (*N* = 696)	2.22 (1.75, 2.82)	< 0.001		2.04 (1.61, 2.60)	< 0.001		1.44 (1.12, 1.86)	0.004	
	Q4 (*N* = 696)	3.54 (2.82, 4.44)	< 0.001		3.10 (2.47, 3.90)	< 0.001		1.69 (1.32, 2.17)	< 0.001	
Continuous PLR		1.01 (1.00, 1.01)	< 0.001		1.00 (1.00, 1.01)	< 0.001		1.00 (1.00, 1.00)	0.243	
Quartiles of PLR	Q1 (*N* = 696)	*Ref*		< 0.001	*Ref*		< 0.001	*Ref*		0.102
	Q2 (*N* = 696)	1.30 (1.02, 1.65)	< 0.034		1.21 (0.95, 1.54)	0.115		1.05 (0.82, 1.34)	0.700	
	Q3 (*N* = 696)	1.72 (1.37, 2.16)	< 0.001		1.68 (1.34, 2.12)	< 0.001		1.28 (1.00, 1.63)	0.047	
	Q4 (*N* = 696)	2.45 (1.97, 3.04)	< 0.001		2.10 (1.69, 2.61)	< 0.001		1.24 (0.98, 1.57)	0.076	
Continuous MLR		1.31 (1.24, 1.37)	< 0.001		1.27 (1.21, 1.34)	< 0.001		1.08 (1.02, 1.15)	0.015	
Quartiles of MLR	Q1 (*N* = 698)	*Ref*		< 0.001	*Ref*		< 0.001	*Ref*		0.099
	Q2 (*N* = 694)	1.14 (0.88, 1.47)	0.312		1.07 (0.83, 1.38)	0.615		0.86 (0.66, 1.12)	0.260	
	Q3 (*N* = 696)	1.90 (1.51, 2.40)	< 0.001		1.62 (1.28, 2.04)	< 0.001		1.07 (0.84, 1.38)	0.570	
	Q4 (*N* = 696)	3.00 (2.41, 3.74)	< 0.001		2.44 (1.96, 3.54)	< 0.001		1.13 (0.89, 1.44)	0.303	
Continuous RPR		5.70 (3.28, 9.91)	< 0.001		4.17 (2.35, 7.40)	< 0.001		2.15 (1.06, 4.35)	0.034	
Quartiles of RPR	Q1 (*N* = 698)	*Ref*		< 0.001	*Ref*		< 0.001	*Ref*		0.093
	Q2 (*N* = 694)	1.26 (1.00, 1.58)	0.047		1.33 (1.06, 1.67)	0.015		1.28 (0.97, 1.69)	0.085	
	Q3 (*N* = 696)	1.14 (0.90, 1.43)	0.282		1.10 (0.87, 1.39)	0.419		0.85 (0.66, 1.09)	0.192	
	Q4 (*N* = 696)	2.24 (1.82, 2.75)	< 0.001		1.93 (1.57, 2.38)	< 0.001		0.81 (0.61, 1.08)	0.148	
Continuous SII		1.00 (1.00, 1.00)	< 0.001		1.00 (1.00, 1.00)	< 0.001		1.00 (1.00, 1.00)	0.002	
Quartiles of SII	Q1 (*N* = 696)	*Ref*		< 0.001	*Ref*		< 0.001	*Ref*		< 0.001
	Q2 (*N* = 696)	0.95 (0.75, 1.21)	0.684		0.94 (0.73, 1.20)	0.605		1.14 (0.88, 1.47)	0.314	
	Q3 (*N* = 696)	1.49 (1.19, 1.86)	< 0.001		1.42 (1.14, 1.77)	0.002		1.21 (0.96, 1.54)	0.113	
	Q4 (*N* = 696)	2.49 (2.02, 3.06)	< 0.001		2.24 (1.82, 2.75)	< 0.001		1.73 (1.34, 2.22)	< 0.001	
Continous SIRI		1.00 (1.00, 1.01)	< 0.001		1.00 (1.00, 1.01)	< 0.001		1.00 (1.00, 1.00)	0.019	
Quartiles of SIRI	Q1 (*N* = 696)	*Ref*		< 0.001	*Ref*		< 0.001	*Ref*		< 0.001
	Q2 (*N* = 696)	1.28 (0.99, 1.64)	0.056		1.17 (0.91, 1.51)	0.218		1.01 (0.78, 1.31)	0.936	
	Q3 (*N* = 696)	2.59 (2.07, 3.25)	< 0.001		2.19 (1.74, 2.75)	< 0.001		1.49 (1.16, 1.90)	0.002	
	Q4 (*N* = 696)	2.45 (1.95, 3.08)	< 0.001		2.31 (1.84, 2.91)	< 0.001		1.72 (1.32, 2.23)	< 0.001	

Mode I: unadjusted; model II: adjusted by age, sex, heart rate, and oxygen saturation; model III: model II further adjusted by hypertension, atrial fibrillation, congestive heart failure, cerebrovascular disease, kidney disease, liver disease, peripheral vascular disease, malignant tumor, red blood cell counts, white blood cell counts, hemoglobin, platelet counts, creatinine, blood Urea nitrogen, electrolytes, glucose, total bilirubin, plasma prothrombin time, acute physiology score III, Oxford acute severity score, Sepsis-Related organ failure assessment score, systemic inflammatory response score system, coronary artery bypass grafting, aspirin, Statins, β-blockers, angiotensin converting enzyme inhibitors/angiotensin receptor blockers, calcium channel blockers, nitrates. HR, hazard ratio; CI, confidence interval; Q, quartile; ref, reference; RDW, red blood cell distribution width; NLR, neutrophil to lymphocyte ratio; PLR, platelet to lymphocyte ratio; MLR, monocyte to lymphocyte ratio; RPR, red blood cell volume distribution width to platelet ratio; SII, systemic immune inflammation index; SIRI, systemic inflammatory response index.

RCS curves showed a nonlinear relationship between 90-day mortality and inflammatory markers in AMI patients (*P* < 0.001). The risk of death increased with elevated inflammatory markers (Fig. 2C). Kaplan-Meier survival curves (Fig. 3) illustrated a decrease in the 90-day survival probability of patients as the quartiles of inflammatory markers increased (*P* < 0.001). Among them, the differences in predicting patient mortality rates after about 90 days gradually diminished between Q3 and Q4 of the inflammatory marker SIRI.

### Efficacy of inflammatory markers to predict in-hospital death

**Fig. 4:**
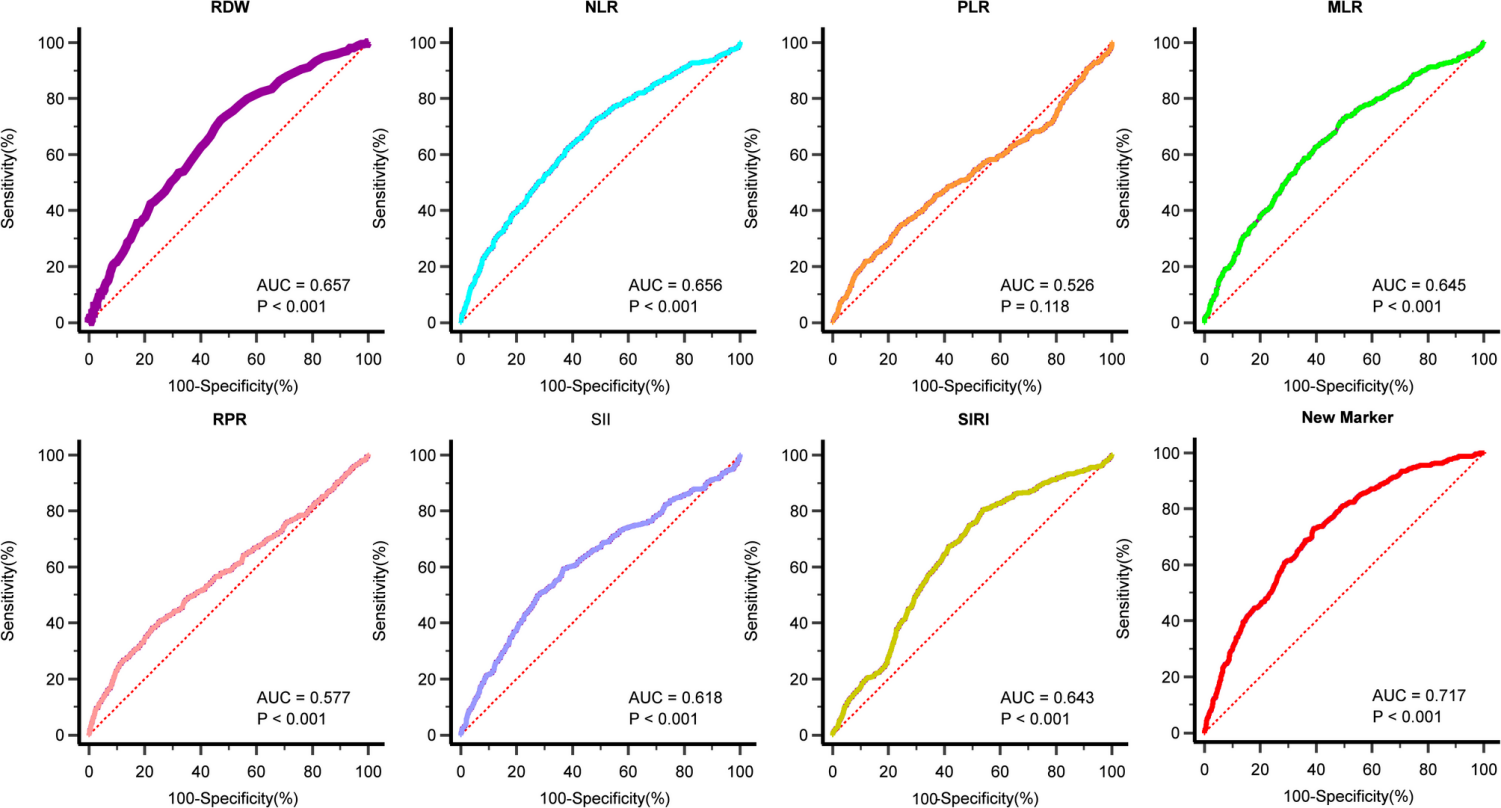
The receiver operator characteristic curves for inflammation indicators as well as a combined new marker for predicting in-hospital mortality respectively. AUC, area under the curve; RDW, red blood cell distribution width; NLR, neutrophil to lymphocyte ratio; PLR, platelet to lymphocyte ratio; MLR, monocyte to lymphocyte ratio; RPR, red blood cell volume distribution width to platelet ratio; SII, systemic immune inflammation index; SIRI, systemic inflammatory response index.

ROC analysis demonstrated that the AUC for predicting in-hospital mortality AMI was significantly higher for the novel combined marker than for other inflammatory markers (New marker: *Z* = 16.712, *P* < 0.001; RDW: *Z* = 11.117, *P* < 0.001; NLR: *Z* = 10.555, *P* < 0.001; PLR: *Z* = 1.563, *P* = 0.118; MLR: *Z* = 9.811, *P* < 0.001; RPR: *Z* = 4.726, *P* < 0.001; SII: *Z* = 7.461, *P* < 0.001; SIRI: *Z* = 10.243, *P* < 0.001). The AUC for the novel marker was 0.720, with a sensitivity of 72.62% and a specificity of 60.19% (Fig. 4). Except for PLR, all other markers exhibited significant predictive value for in-hospital mortality in AMI (*P* < 0.001).

### Correlation analysis between inflammatory markers and CRP

After removing patients with missing CRP markers, we did Spearman’s correlation analysis of inflammatory markers and CRP in 242 AMI patients, which showed that CRP was positively correlated with NLR (*r* = 0.18, *P* = 0.004), PLR (*r* = 0.19, *P* = 0.003), MLR (*r* = 0.19, *P* = 0.003), and SII (*r* = 0.18, *P* = 0.004) were positively correlated.

### Sensitivity analysis

To evaluate the potential impact of sepsis and autoimmune diseases on the study results, we included patients with a history of these comorbidities (sepsis, *n* = 457; autoimmune disease, *n* = 20) and re-analyzed the data (Supplementary Table S2). Compared to Q1, Q4 of RDW was an independent risk factor for 90-day mortality among AMI patients (HR 1.42, *P* = 0.048). Compared to Q1, Q4 of NLR were independent risk factors for in-hospital mortality (OR 1.90, *P* = 0.022), 30-day mortality (HR 2.03, *P* < 0.001), and 90-day mortality (HR 1.81, *P* < 0.001). Compared to Q1, Q4 of SII were independent risk factors for in-hospital mortality (OR 2.04, *P* = 0.012), 30-day mortality (HR 2.02, *P* < 0.001), and 90-day mortality (HR 1.76, *P* < 0.001). Compared to Q1, Q4 of SIRI were independent risk factors for in-hospital mortality (OR 1.97, *P* = 0.021), 30-day mortality (HR 2.17, *P* < 0.001), and 90-day mortality (HR 1.59, *P* = 0.005).

To evaluate the potential impact of malignant cancer and renal disease on the study results, we excluded patients with a history of these conditions (*n* = 1068) and re-analyzed the data (Supplementary Table S3). Compared to Q1, Q4 of NLR were independent risk factors for in-hospital mortality, 30-day and 90-day mortality (OR 1.85, *P* = 0.023; HR 1.76, *P* = 0.016; HR 1.92, *P* = 0.001). Compared to Q1, Q4 of SII were independent risk factors for in-hospital mortality, 30-day and 90-day mortality (OR 1.88, *P* = 0.040; HR 1.54, *P* = 0.040; HR 1.69, *P* = 0.008). Compared to Q1, Q4 of SIRI were independent risk factors for hospital mortality, 30-day and 90-day mortality (OR 2.28, *P* = 0.019; HR 1.64, *P* = 0.032; HR 1.51, *P* = 0.036).

To evaluate the potential impact of COPD on the study results, we excluded patients with a history of COPD (*n* = 665) and re-analyzed the data (Supplementary Table S4). Compared to Q1, Q4 of RDW (OR 2.37, *P* = 0.003) and SIRI (OR 1.74, *P* = 0.036) were independent risk factors for in-hospital mortality; compared to Q1, Q4 of RDW (HR 1.51, *P* = 0.041), SII (HR 1.80, *P* = 0.001) and SIRI (HR 1.65, *P* = 0.008) were independent risk factors for 30-day mortality; compared to Q1, Q4 of SII (HR 1.76, *P* < 0.001) and SIRI (HR 1.59, *P* = 0.005) were independent risk factors for 90-day mortality.

## Discussion

Study assessed associations between hematological inflammatory indices (RDW, NLR, PLR, MLR, RPR, SII, SIRI) and mortality in acute myocardial infarction (AMI) patients. Consistent with prior evidence, elevated RDW, NLR, SII, and SIRI were independently associated with increased all-cause mortality in AMI^2,16^. Immune cells, including lymphocytes, neutrophils, and monocytes, play critical roles in coronary atherosclerosis progression. Although inflammatory responses are increasingly linked to coronary heart disease, the prognostic significance of specific inflammatory markers in AMI remains underexplored. Persistent proinflammatory states may exacerbate post-MI left ventricular remodeling, highlighting inflammation as a potential therapeutic target for improving AMI outcomes.

Previously, studies have utilized inflammatory markers such as C-reactive protein, white blood cell counts, Tumor Necrosis Factor-alpha, interleukins, and hs-CRP to assess inflammation in myocardial infarction, which is closely associated with cardiovascular event risk, disease severity, and prognosis^17^. Routine blood tests, which are cost-effective and widely accessible, have gained increasing attention for evaluating inflammatory markers in peripheral blood and their relationship with cardiovascular mortality^18^. While traditionally used in diagnosing anemia, RDW has been linked to various conditions, including heart failure, atrial fibrillation, stroke, and dyslipidemia. The Malmö Diet and Cancer Study found that individuals in the highest RDW quartile had an elevated risk of fatal acute coronary events over a 14-year follow-up^19^. Similarly, a study of 312 STEMI patients undergoing thrombolysis demonstrated that baseline RDW independently predicted ST-segment regression and MACE^20^. This association may stem from reduced erythrocyte deformability and impaired microvascular perfusion, exacerbating microcirculatory dysfunction in AMI^21^.

Elevated neutrophil levels are associated with increased risks of ischemic events and AMI mortality^22^. Núñez et al. study demonstrated that post-hospitalization NLR independently predicts 1-year reinfarction and mortality in AMI patients with diabetes^23^. Consistent with these findings, our study identified NLR as an independent risk factor for in-hospital, 30-day and 90-day mortality in AMI, with higher values correlating with increased mortality risk, likely due to neutrophils exacerbating inflammatory responses during myocardial repair and left ventricular remodeling^24^. Meta-analyses suggest that elevated PLR levels are associated with increased risks of MACE, including cardiovascular mortality, MI, and ischemic stroke, in ACS populations^25^. Previous studies have shown heterogeneity in PLR’s predictive value and cutoff thresholds^26^, and while its relationship with ACS severity and prognosis exists, its independent predictive role requires further validation.

A positive association between elevated MLR and increased mortality risk in the general population, with MLR serving as a strong independent predictor of cardiovascular and all-cause mortality^27^. The RPR is associated with inflammatory responses and disease severity, particularly in systemic inflammatory response syndrome (SIRS) and sepsis^13^. Wu et al. reported that elevated RPR was significantly associated with sepsis and increased mortality in acute kidney injury patients^28^. However, our study RPR have not shown that this index would be predictive of hospital mortality in AMI, this discrepancy may be attributed to differences in the baseline characteristics, comorbidities, or disease severity of our study population compared to previous studies. Additionally, RPR is a dynamic marker that can fluctuate rapidly in response to acute physiological changes. In our study, RPR was measured only at the time of admission, which may have influenced its association with outcomes. SII and SIRI are novel inflammatory markers that reflect both local and systemic immune responses^29,30^. SII is positively correlated with myocardial injury markers and accurately predicts short- and long-term major adverse cardiovascular events (MACE) in ACS patients^31^. 20-year cohort study of 42,875 US adults demonstrated that an SII level > 655.56 was associated with higher cardiovascular mortality (HR = 1.33; 95% CI: 1.11–1.59) compared to an SII level < 335.36^32^. Similarly, SIRI, a marker of systemic inflammation, indicated that adults with SIRI > 1.43 had a higher risk of cardiovascular mortality (HR = 1.39; 95% CI: 1.14–1.68) than those with SIRI < 0.68^32^. Both SII and SIRI have significant clinical implications for AMI prognosis, although the underlying mechanisms require further investigation. Our study, excluding sepsis and immune diseases, confirms that these inflammatory factors remain independent risk factors for in-hospital and short-term AMI mortality.

Inflammation plays a key role in arrhythmic events and higher short-term mortality in AMI patients^33^. Cytokines like IL-6, TNF-α, and CRP disrupt cardiac function, increase oxidative stress, and promote fibrosis, heightening arrhythmia risk. Inflammation also worsens ischemia-reperfusion injury, further destabilizing the myocardium^34^. This link may explain the higher mortality in AMI patients, as arrhythmias are a major cause of death^35^. Targeting inflammatory pathways could reduce arrhythmic events and improve outcomes in AMI^36^. Future research should focus on targeting inflammatory pathways to reduce arrhythmic events and improve AMI outcomes. Furthermore, lipoprotein(a) [Lp(a)] has been increasingly recognized as an independent risk factor for cardiovascular disease, including AMI. Elevated Lp(a) levels are associated with atherosclerosis progression and adverse cardiovascular outcomes, as highlighted by DiFusco et al. Lp(a) promotes inflammation, thrombosis, and plaque instability, contributing to the pathogenesis of AMI^37^.

Our study helps clinicians identify high-risk populations for in-hospital mortality, enabling timely interventions to improve prognosis. However, it has several limitations. First, as a retrospective analysis, it relied on pre-existing data, which may introduce biases such as incomplete records, missing information, or inconsistencies in data collection. The lack of control over variables and the inability to establish causality are inherent challenges in retrospective designs. Second, although we adjusted for multiple confounding factors, unmeasured variables and traditional inflammatory markers not evaluated due to data limitations may still influence in-hospital mortality. Third, we only included patients diagnosed with AMI during hospitalization, excluding initial admission diagnoses. We did not analyze STEMI and NSTEMI subgroups separately or assess the impact of left ventricular function, Killip class, or cardiogenic shock, which are limitations of the MIMIC-IV database. Future large-scale prospective trials should incorporate these clinical characteristics for further analysis. Finally, although our novel biomarker has demonstrated good predictive ability for in-hospital mortality, direct comparisons with validated clinical scores such as GRACE and TIMI were not performed due to limitations of our database. Future prospective studies should be conducted to evaluate its additive and comparative performance against these established risk stratification tools.

## Conclusion

RDW, NLR, SII, and SIRI were independent risk factors for in-hospital, 30-day, and 90-day mortality in AMI patients. Elevated levels of these indicators highlight the role of inflammation in AMI prognosis. Monitoring them could improve risk assessment and patient management.

## Electronic supplementary material

Below is the link to the electronic supplementary material.

Supplementary Material 1

## Data Availability

The datasets used and/or analyzed during the current study are available from the corresponding author on reasonable request.

## Abbreviations

AMI: Acute myocardial infarction
AUC: Area under the curve
STEMI: ST-segment elevation myocardial infarction
USTEMI: Non ST-segment elevation myocardial infarction
NLR: Neutrophil count to lymphocyte count ratio
PLR: Platelet count to lymphocyte count ratio
MLR: Monocyte count to lymphocyte count ratio
RDW: Red blood cell distribution width
RPR: Red blood cell volume distribution width to platelet count
ICU: Intensive care unit
SII: Systemic immune inflammation index
SIRI: Systemic inflammatory response index
CVD: Cardiovascular disease
MIMIC-IV: Medical Information Mart for Intensive Care-IV
ICD: International Classification of Diseases
BMI: Body mass index
COPD: Chronic obstructive pulmonary disease
RBC: Red blood cell counts
WBC: White blood cell counts
BUN: Blood urea nitrogen
PT: Plasma prothrombin time
APTT: Activated partial prothrombin time
APSIII: Acute Physiology Score III
OASIS: Oxford Acute Severity Score
SOFA: Sepsis-Related Organ Failure Assessment Score
SIRS: Systemic Inflammatory Response Syndrome
PCI: Percutaneous coronary intervention
CABG: Coronary artery bypass grafting
BBs: β-Blockers
ACEI/ARBs: Angiotensin converting enzyme inhibitors/angiotensin receptor blockers
CCB: Calcium channel blockers
OR: Odds ratio
HR: Hazard ratio
CI: Confidence interval
ROC: Receiver operating characteristic
Hs-CRP: High-sensitivity C-reactive protein
SD: Standard deviation
MCV: Mean red blood cell counts cell volume
MACE: Major adverse cardiac events
