# Supplementary material for: The effect of inflammatory markers on mortality in patients with acute myocardial infarction
Source: Sci Rep. 2025 Apr 25;15:14514. doi: 10.1038/s41598-025-98408-y (PMC12032369; doi:10.1038/s41598-025-98408-y)
Supplement: Supplementary file 1 — Supplementary Material 1 [file 41598_2025_98408_MOESM1_ESM.docx]

**Supplementary Table S1.** ICD code.

**Supplementary Table S2.** Sensitivity analysis of three outcomes after adjusting sepsis and autoimmune.

**Supplementary Table S3.** Sensitivity analysis of three outcomes after removal of renal disease and malignant cancer. **Supplementary Table S4.** Sensitivity analysis of three outcomes after removal of COPD.

**Supplementary Figure 1.** Analysis of the Correlation Between Inflammatory Markers and CRP

**Supplementary Table S1.** ICD code. ICD, International Classification of Diseases; AMI, acute myocardial infarction; CABG, coronary artery bypass grafting; PCI, percutaneous coronary intervention.

| Category | ICD code |
| --- | --- |
| AMI | '41000','41001','41002','41010','41011','41012','41020','41021','41022','41030','41031','41032','41040','41041','41042','41050','41051','41052','41080','41081','41082','41090','41091','41092','I21','I210','I2101','I2102','I2109','I211','I2111','I2119','I212','I2121','I2129','I213','I214','I219','I21A','I21A1','I21A9','I220','I221','I222','I228','I229','I23','I230','I231','I232','I233','I234','I235', 'I236','I238' |
| CABG | '210083','210088','210089','021008C','021008F','021008W','210093','210098','210099','021009C','021009F','021009W','02100A3','02100A8','02100A9','02100AC','02100AF','02100AW','02100J3','02100J8','02100J9','02100JC','02100JF','02100JW','02100K3','02100K8','02100K9','02100KC','02100KF','02100KW','02100Z3','02100Z8','02100Z9','02100ZC','02100ZF','210344','02103D4','210444','210483','210488','210489','021048C','021048F','021048W','210493','210498','210499','021049C','021049F','021049W','02104A3','02104A8','02104A9','02104AC','02104AF','02104AW','02104D4','02104J3','02104J8','02104J9','02104JC','02104JF','02104JW','02104K3','02104K8','02104K9','02104KC','02104KF','02104KW','02104Z3','02104Z8','02104Z9','02104ZC','02104ZF','211083','211088','211089','021108C','021108F','021108W','211093','211098','211099','021109C','021109F','021109W','02110A3','02110A8','02110A9','02110AC','02110AF','02110AW','02110J3','02110J8','02110J9','02110JC','02110JF','02110JW','02110K8','02110K9','02110KC','02110KF','02110KW','02110Z3','02110Z8','02110Z9','02110ZC','02110ZF','211344','02113D4','211444','211483','211488','211489','021148C','021148F','021148W','211493','211498','211499','021149C','021149F','021149W','02114A3','02114A8','02114A9','02114AC','02114AF','02114AW','02114D4','02114J3','02114J8','02114J9','02114JC','02114JF','02114JW','02114K3','02114K8','02114K9','02114KC','02114KF','02114KW','02114Z3','02114Z8','02114Z9','02114ZC','02114ZF','212083','212088','212089','021208C','021208F','021208W','212093','212098','212099','021209C','021209F','021209W','02120A3','02120A8','02120A9','02120AC','02120AF','02120AW','02120J3','02120J8','02120J9','02120JC','02120JF','02120JW','02120K3','02120K8','02120K9','02120KC','02120KF','02120KW','02120Z3','02120Z8','02120Z9','02120ZC','02120ZF','212344','02123D4','212444','212483','212488','212489','021248C','021248F','021248W','212493','212498','212499','021249C','021249F','021249W','02124A3','02124A8','02124A9','02124AC','02124AF','02124AW','02124D4','02124J3','02124J8','02124J9','02124JC','02124JF','02124JW','02124K3','02124K8','02124K9','02124KC','02124KF','02124KW','02124Z3','02124Z8','02124Z9','02124ZC','02124ZF','213083','213088','213089','021308C','021308F','021308W','213093','213098','213099','021309C','021309F','021309W','02130A3','02130A8','02130A9','02130AC','02130AF','02130AW','02130J3','02130J8','02130J9','02130JC','02130JF','02130JW','02130K3','02130K8','02130K9','02130KC','02130KF','02130KW','02130Z3','02130Z8','02130Z9','02130ZC','02130ZF','213344','02133D4','213444','213483','213488','213489','021348C','021348F','021348W','213493','213498','213499','021349C','021349F','021349W','02134A3','02134A8','02134A9','02134AC','02134AF','02134AW','02134D4','02134J3','02134J8','02134J9','02134JC','02134JF','02134JW','02134K3','02134K8','02134K9','02134KC','02134KF','02134KW','02134Z3','02134Z8','02134Z9','02134ZC','02134ZF' |
| PCI | '02703DZ','02703ZZ','02713DZ','02713ZZ','02723DZ','02723ZZ','02733DZ','02733ZZ' |

**Supplementary Table S2.** Sensitivity analysis of three outcomes after adjusting sepsis and autoimmune. Adjusted by age, sex, heart rate, and oxygen saturation, hypertension, atrial fibrillation, congestive heart failure, cerebrovascular disease, kidney disease, liver disease, peripheral vascular disease, malignant tumor, red blood cell counts, white blood cell counts, hemoglobin, platelet counts, creatinine, blood urea nitrogen, electrolytes, glucose, total bilirubin, plasma prothrombin time, Acute Physiology Score III, Oxford Acute Severity Score, Sepsis-Related Organ Failure Assessment Score, Systemic Inflammatory Response Score System, coronary artery bypass grafting, aspirin, statins, β-blockers, angiotensin converting enzyme inhibitors/angiotensin receptor blockers, calcium channel blockers, nitrates. OR, odds ratio; HR, hazard ratio; CI, confidence interval; Q, quartile; Ref, reference; RDW, red blood cell distribution width; NLR, neutrophil to lymphocyte ratio; PLR, platelet to lymphocyte ratio; MLR, monocyte to lymphocyte ratio; RPR, red blood cell volume distribution width to platelet ratio; SII, systemic immune inflammation index; SIRI, systemic inflammatory response index.

| **Category** | | **Hospital mortality** | | | **30-day mortality** | | | **90-day mortality** | | |
| --- | --- | --- | --- | --- | --- | --- | --- | --- | --- | --- |
|  |  | ***OR (95% CI)*** | ***P*** | ***P for trend*** | ***HR (95% CI)*** | ***P*** | ***P for trend*** | ***HR (95% CI)*** | ***P*** | ***P for trend*** |
| RDW | Q1 (N=734) | *Ref* |  | 0.014 | *Ref* |  | 0.098 | *Ref* |  | 0.016 |
|  | Q2 (N=687) | 0.73 (0.41, 1.31) | 0.302 |  | 0.86 (0.59, 1.26) | 0.429 |  | 0.91 (0.65, 1.27) | 0.577 |  |
|  | Q3 (N=662) | 1.53 (0.88, 2.67) | 0.132 |  | 1.26 (0.86, 1.84) | 0.233 |  | 1.33 (0.96, 1.85) | 0.085 |  |
|  | Q4 (N=701) | 1.67 (0.92, 3.06) | 0.095 |  | 1.28 (0.86, 1.92) | 0.229 |  | 1.42 (1.00, 2.02) | 0.048 |  |
| NLR | Q1 (N=696) | *Ref* |  | 0.058 | *Ref* |  | 0.001 | *Ref* |  | 0.001 |
|  | Q2 (N=696) | 1.54 (0.87, 2.72) | 0.138 |  | 1.38 (0.92, 2.08) | 0.121 |  | 1.35 (0.98, 1.87) | 0.069 |  |
|  | Q3 (N=696) | 2.05 (1.19, 3.52) | 0.009 |  | 1.87 (1.27, 2.73) | 0.001 |  | 1.62 (1.19, 2.21) | 0.002 |  |
|  | Q4 (N=696) | 1.90 (1.10, 3.29) | 0.022 |  | 2.03 (1.39, 2.97) | < 0.001 |  | 1.81 (1.33, 2.48) | < 0.001 |  |
| PLR | Q1 (N=696) | *Ref* |  | 0.393 | *Ref* |  | 0.123 | *Ref* |  | 0.223 |
|  | Q2 (N=696) | 0.77 (0.46, 1.34) | 0.341 |  | 0.83 (0.58, 1.17) | 0.275 |  | 0.88 (0.65, 1.18) | 0.377 |  |
|  | Q3 (N=696) | 0.75 (0.46, 1.22) | 0.246 |  | 0.68 (0.48, 0.96) | 0.029 |  | 0.78 (0.59, 1.05) | 0.098 |  |
|  | Q4 (N=696) | 1.06 (0.67, 1.69) | 0.798 |  | 0.94 (0.69, 1.30) | 0.708 |  | 1.01 (0.77, 1.32) | 0.944 |  |
| MLR | Q1 (N=698) | *Ref* |  | 0.202 | *Ref* |  | 0.293 | *Ref* |  | 0.555 |
|  | Q2 (N=694) | 1.26 (0.71, 2.25) | 0.427 |  | 0.95 (0.65, 1.39) | 0.785 |  | 0.89 (0.64, 1.22) | 0.451 |  |
|  | Q3 (N=696) | 1.70 (1.00, 2.91) | 0.052 |  | 1.18 (0.83, 1.68) | 0.362 |  | 1.06 (0.79, 1.43) | 0.682 |  |
|  | Q4 (N=696) | 1.62 (0.95, 2.76) | 0.438 |  | 1.27 (0.90, 1.80) | 0.181 |  | 1.07 (0.80, 1.43) | 0.653 |  |
| RPR | Q1 (N=698) | *Ref* |  | 0.212 | *Ref* |  | 0.212 | *Ref* |  | 0.257 |
|  | Q2 (N=694) | 0.93 (0.52, 1.66) | 0.812 |  | 0.88 (0.59, 1.29) | 0.506 |  | 0.85 (0.60, 1.19) | 0.332 |  |
|  | Q3 (N=696) | 0.58 (0.29, 1.18) | 0.135 |  | 0.63 (0.40, 1.00) | 0.048 |  | 0.69 (0.46, 1.02) | 0.064 |  |
|  | Q4 (N=696) | 0.86 (0.36, 2.05) | 0.726 |  | 0.67 (0.38, 1.18) | 0.163 |  | 0.77 (0.48, 1.26) | 0.306 |  |
| SII | Q1 (N=696) | *Ref* |  | 0.028 | *Ref* |  | < 0.001 | *Ref* |  | 0.001 |
|  | Q2 (N=696) | 1.05 (0.61, 1.82) | 0.850 |  | 1.11 (0.76, 1.63) | 0.588 |  | 1.07 (0.78, 1.47) | 0.642 |  |
|  | Q3 (N=696) | 1.22 (0.72, 2.06) | 0.463 |  | 1.24 (0.86, 1.79) | 0.248 |  | 1.26 (0.94, 1.70) | 0.125 |  |
|  | Q4 (N=696) | 2.04 (1.19, 3.57) | 0.012 |  | 2.02 (1.39, 2.95) | < 0.001 |  | 1.76 (1.28, 2.42) | < 0.001 |  |
| SIRI | Q1 (N=696) | *Ref* |  | 0.029 | *Ref* |  | < 0.001 | *Ref* |  | 0.002 |
|  | Q2 (N=696) | 1.15 (0.65, 2.03) | 0..637 |  | 1.09 (0.72, 1.64) | 0.685 |  | 0.97 (0.71, 1.33) | 0.872 |  |
|  | Q3 (N=696) | 1.74 (1.00, 3.02) | 0.051 |  | 1.78 (1.20, 2.64) | 0.004 |  | 1.40 (1.03, 1.91) | 0.034 |  |
|  | Q4 (N=696) | 1.97 (1.11, 3.49) | 0.021 |  | 2.17 (1.45, 3.24) | < 0.001 |  | 1.59 (1.15, 2.20) | 0.005 |  |

**Supplementary Table S3.** Sensitivity analysis of three outcomes after removal of renal disease and malignant cancer. Adjusted by age, sex, heart rate, and oxygen saturation, hypertension, atrial fibrillation, congestive heart failure, cerebrovascular disease, liver disease, peripheral vascular disease, red blood cell counts, white blood cell counts, hemoglobin, platelet counts, creatinine, blood urea nitrogen, electrolytes, glucose, total bilirubin, plasma prothrombin time, Acute Physiology Score III, Oxford Acute Severity Score, Sepsis-Related Organ Failure Assessment Score, Systemic Inflammatory Response Score System, coronary artery bypass grafting, aspirin, statins, β-blockers, angiotensin converting enzyme inhibitors/angiotensin receptor blockers, calcium channel blockers, nitrates. OR, odds ratio; HR, hazard ratio; CI, confidence interval; Q, quartile; Ref, reference; RDW, red blood cell distribution width; NLR, neutrophil to lymphocyte ratio; PLR, platelet to lymphocyte ratio; MLR, monocyte to lymphocyte ratio; RPR, red blood cell volume distribution width to platelet ratio; SII, systemic immune inflammation index; SIRI, systemic inflammatory response index.

| **Category** | | **Hospital mortality** | | | **30-day mortality** | | | **90-day mortality** | | |
| --- | --- | --- | --- | --- | --- | --- | --- | --- | --- | --- |
|  |  | ***OR (95% CI)*** | ***P*** | ***P for trend*** | ***HR (95% CI)*** | ***P*** | ***P for trend*** | ***HR (95% CI)*** | ***P*** | ***P for trend*** |
| RDW | Q1 (N=455) | *Ref* |  | 0.144 | *Ref* |  | 0.240 | *Ref* |  | 0.521 |
|  | Q2 (N=418) | 0.70 (0.35, 1.40) | 0.316 |  | 0.72 (0.45, 1.15) | 0.166 |  | 0.91 (0.61, 1.37) | 0.670 |  |
|  | Q3 (N=418) | 1.17 (0.62, 2.21) | 0.636 |  | 0.89 (0.57, 1.38) | 0.603 |  | 0.94 (0.63, 1.38) | 0.753 |  |
|  | Q4 (N=425) | 1.26 (0.64, 2.48) | 0.499 |  | 1.07 (0.68, 1.68) | 0.758 |  | 1.14 (0.76, 1.72) | 0.521 |  |
| NLR | Q1 (N=429) | *Ref* |  | 0.058 | *Ref* |  | 0.031 | *Ref* |  | 0.007 |
|  | Q2 (N=429) | 0.94 (0.51, 1.74) | 0.851 |  | 1.05 (0.63, 1.75) | 0.863 |  | 1.29 (0.84, 1.97) | 0.241 |  |
|  | Q3 (N=429) | 1.50 (0.86, 2.62) | 0.148 |  | 1.46 (0.92, 2.32) | 0.107 |  | 1.45 (0.97, 2.18) | 0.066 |  |
|  | Q4 (N=429) | 1.85 (1.10, 3.15) | 0.023 |  | 1.76 (1.11, 2.79) | 0.016 |  | 1.92 (1.29, 2.87) | 0.001 |  |
| PLR | Q1 (N=429) | *Ref* |  | 0.095 | *Ref* |  | 0.123 | *Ref* |  | 0.046 |
|  | Q2 (N=429) | 0.49 (0.26, 0.94) | 0.062 |  | 0.69 (0.45, 1.06) | 0.095 |  | 0.66 (0.45, 1.07) | 0.318 |  |
|  | Q3 (N=429) | 0.54 (0.31, 1.06) | 0.125 |  | 0.56 (0.38, 0.81) | 0.215 |  | 0.64 (0.46, 1.01) | 0.091 |  |
|  | Q4 (N=429) | 0.61 (0.36, 1.12) | 0.131 |  | 0.66 (0.47, 0.95) | 0.123 |  | 0.75 (0.55, 1.06) | 0.057 |  |
| MLR | Q1 (N=429) | *Ref* |  | 0.011 | *Ref* |  | 0.469 | *Ref* |  | 0.092 |
|  | Q2 (N=429) | 1.53 (0.73, 3.23) | 0.263 |  | 0.90 (0.56, 1.45) | 0.671 |  | 0.75 (0.49, 1.13) | 0.174 |  |
|  | Q3 (N=429) | 2.92 (1.46, 5.83) | 0.002 |  | 1.21 (0.78, 1.86) | 0.401 |  | 1.02 (0.69, 1.48) | 0.935 |  |
|  | Q4 (N=429) | 2.31 (1.18, 4.54) | 0.015 |  | 1.18 (0.77, 1.79) | 0.435 |  | 1.16 (0.81, 1.67) | 0.405 |  |
| RPR | Q1 (N=429) | *Ref* |  | 0.332 | *Ref* |  | 0.219 | *Ref* |  | 0.260 |
|  | Q2 (N=429) | 0.55 (0.27, 1.09) | 0.087 |  | 0.58 (0.36, 0.93) | 0.124 |  | 0.61 (0.43, 1.21) | 0.170 |  |
|  | Q3 (N=429) | 0.55 (0.25, 1.21) | 0.140 |  | 0.47 (0.28, 0.78) | 0.312 |  | 0.51 (0.33, 1.01) | 0.400 |  |
|  | Q4 (N=429) | 0.44 (0.17, 1.16) | 0.098 |  | 0.43 (0.23, 0.81) | 0.118 |  | 0.44 (0.25, 0.78) | 0.053 |  |
| SII | Q1 (N=429) | *Ref* |  | 0.042 | *Ref* |  | 0.012 | *Ref* |  | 0.021 |
|  | Q2 (N=429) | 1.07 (0.56, 2.01) | 0.845 |  | 1.03 (0.65, 1.64) | 0.891 |  | 1.15 (0.77, 1.69) | 0.488 |  |
|  | Q3 (N=429) | 1.22 (0.66, 2.26) | 0.517 |  | 1.13 (0.73, 1.75) | 0.581 |  | 1.17 (0.80, 1.71) | 0.401 |  |
|  | Q4 (N=429) | 1.88 (1.23, 3.61) | 0.040 |  | 1.54 (1.03, 2.42) | 0.040 |  | 1.69 (0.15, 2.51) | 0.008 |  |
| SIRI | Q1 (N=429) | *Ref* |  | 0.068 | *Ref* |  | 0.012 | *Ref* |  | 0.008 |
|  | Q2 (N=429) | 1.18 (0.60, 2.30) | 0.628 |  | 0.94 (0.58, 1.51) | 0.796 |  | 0.87 (0.58, 1.30) | 0.504 |  |
|  | Q3 (N=429) | 1.43 (0.75, 2.73) | 0.271 |  | 1.08 (0.68, 1.69) | 0.754 |  | 1.06 (0.72, 1.55) | 0.761 |  |
|  | Q4 (N=429) | 2.28 (1.14, 4.56) | 0.019 |  | 1.64 (1.05, 2.57) | 0.032 |  | 1.51 (1.03, 2.23) | 0.036 |  |

**Supplementary Table S4.** Sensitivity analysis of three outcomes after removal of COPD. Adjusted by age, sex, heart rate, and oxygen saturation, hypertension, atrial fibrillation, congestive heart failure, cerebrovascular disease, kidney disease, liver disease, peripheral vascular disease, malignant tumor, red blood cell counts, white blood cell counts, hemoglobin, platelet counts, creatinine, blood urea nitrogen, electrolytes, glucose, total bilirubin, plasma prothrombin time, Acute Physiology Score III, Oxford Acute Severity Score, Sepsis-Related Organ Failure Assessment Score, Systemic Inflammatory Response Score System, coronary artery bypass grafting, aspirin, statins, β-blockers, angiotensin converting enzyme inhibitors/angiotensin receptor blockers, calcium channel blockers, nitrates. OR, odds ratio; HR, hazard ratio; CI, confidence interval; Q, quartile; Ref, reference; RDW, red blood cell distribution width; NLR, neutrophil to lymphocyte ratio; PLR, platelet to lymphocyte ratio; MLR, monocyte to lymphocyte ratio; RPR, red blood cell volume distribution width to platelet ratio; SII, systemic immune inflammation index; SIRI, systemic inflammatory response index; COPD, chronic obstructive pulmonary disease.

| **Category** | | **Hospital mortality** | | | **30-day mortality** | | | **90-day mortality** | | |
| --- | --- | --- | --- | --- | --- | --- | --- | --- | --- | --- |
|  |  | ***OR (95% CI)*** | ***P*** | ***P for trend*** | ***HR (95% CI)*** | ***P*** | ***P for trend*** | ***HR (95% CI)*** | ***P*** | ***P for trend*** |
| RDW | Q1 (N=618) | *Ref* |  | 0.005 | *Ref* |  | 0.027 | *Ref* |  | 0.001 |
|  | Q2 (N=452) | 1.14 (0.66, 1.96) | 0.644 |  | 0.92 (0.63, 1.35) | 0.688 |  | 1.07 (0.76, 1.50) | 0.676 |  |
|  | Q3 (N=531) | 1.92 (0.13, 3.25) | 0.016 |  | 1.25 (0.86, 1.81) | 0.232 |  | 1.50 (1.08, 2.07) | 0.014 |  |
|  | Q4 (N=518) | 2.37 (1.34, 4.21) | 0.003 |  | 1.51 (1.02, 2.25) | 0.041 |  | 1.77 (1.25, 2.51) | 0.001 |  |
| NLR | Q1 (N=530) | *Ref* |  | 0.458 | *Ref* |  | 0.041 | *Ref* |  | 0.130 |
|  | Q2 (N=530) | 1.07 (0.65, 1.78) | 0.787 |  | 0.89 (0.65, 1.24) | 0.513 |  | 0.85 (0.64, 1.13) | 0.260 |  |
|  | Q3 (N=530) | 1.32 (0.82, 2.15) | 0.255 |  | 0.70 (0.50, 0.97) | 0.053 |  | 0.80 (0.61, 1.05) | 0.110 |  |
|  | Q4 (N=529) | 1.40 (0.86, 2.25) | 0.170 |  | 1.08 (0.81, 1.46) | 0.589 |  | 1.05 (0.82, 1.36) | 0.677 |  |
| PLR | Q1 (N=530) | *Ref* |  | 0.018 | *Ref* |  | 0.123 | *Ref* |  | 0.130 |
|  | Q2 (N=530) | 0.69 (0.43, 1.10) | 0.119 |  | 0.83 (0.58, 1.17) | 0.275 |  | 0.88 (0.65, 1.18) | 0.377 |  |
|  | Q3 (N=530) | 0.50 (0.31, 0.80) | 0.414 |  | 0.68 (0.48, 1.06) | 0.029 |  | 0.78 (0.59, 1.05) | 0.098 |  |
|  | Q4 (N=529) | 0.91 (0.59, 1.39) | 0.653 |  | 0.94 (0.69, 1.30) | 0.708 |  | 1.01 (0.77, 1.32) | 0.944 |  |
| MLR | Q1 (N=530) | *Ref* |  | 0.555 | *Ref* |  | 0.159 | *Ref* |  | 0.171 |
|  | Q2 (N=530) | 1.08 (0.65, 2.25) | 0.758 |  | 0.78 (0.55, 1.12) | 0.181 |  | 0.83 (0.61, 1.12) | 0.236 |  |
|  | Q3 (N=530) | 1.36 (0.83, 2.22) | 0.219 |  | 1.02 (0.72, 1.42) | 0.925 |  | 1.07 (0.80, 1.43) | 0.628 |  |
|  | Q4 (N=529) | 1.30 (0.80, 2.09) | 0.288 |  | 1.12 (0.80, 1.54) | 0.500 |  | 1.11 (0.83, 1.47) | 0.479 |  |
| RPR | Q1 (N=530) | *Ref* |  | 0.258 | *Ref* |  | 0.021 | *Ref* |  | 0.009 |
|  | Q2 (N=530) | 0.87 (0.52, 1.51) | 0.636 |  | 0.75 (0.52, 1.09) | 0.137 |  | 0.71 (0.51, 0.98) | 0.040 |  |
|  | Q3 (N=530) | 0.56 (0.29, 1.08) | 0.088 |  | 0.53 (0.34, 0.83) | 0.005 |  | 0.52 (0.36, 0.76) | < 0.001 |  |
|  | Q4 (N=529) | 0.72 (0.32, 1.61) | 0.419 |  | 0.45 (0.26, 0.78) | 0.004 |  | 0.49 (0.31, 0.78) | 0.003 |  |
| SII | Q1 (N=530) | *Ref* |  | 0.045 | *Ref* |  | < 0.001 | *Ref* |  | 0.003 |
|  | Q2 (N=530) | 0.89 (0.55, 1.45) | 0.644 |  | 1.04 (0.72, 1.48) | 0.841 |  | 1.11 (0.83, 1.50) | 0.473 |  |
|  | Q3 (N=530) | 0.97 (0.61, 1.56) | 0.908 |  | 1.12 (0.80, 1.58) | 0.492 |  | 1.19 (0.89, 1.58) | 0.231 |  |
|  | Q4 (N=529) | 1.64 (0.98, 2.72) | 0.058 |  | 1.80 (1.26, 2.55) | 0.001 |  | 1.68 (1.24, 2.28) | < 0.001 |  |
| SIRI | Q1 (N=530) | *Ref* |  | 0.034 | *Ref* |  | 0.006 | *Ref* |  | 0.002 |
|  | Q2 (N=530) | 1.02 (0.61, 1.72) | 0.932 |  | 1.01 (0.69, 1.48) | 0.946 |  | 1.08 (0.79, 1.47) | 0.615 |  |
|  | Q3 (N=530) | 1.70 (1.05, 2.74) | 0.031 |  | 1.52 (1.08, 2.15) | 0.017 |  | 1.57 (1.18, 2.09) | 0.002 |  |
|  | Q4 (N=529) | 1.74 (1.04, 2.93) | 0.036 |  | 1.65 (1.14, 2.38) | 0.008 |  | 1.57 (1.15, 2.15) | 0.005 |  |


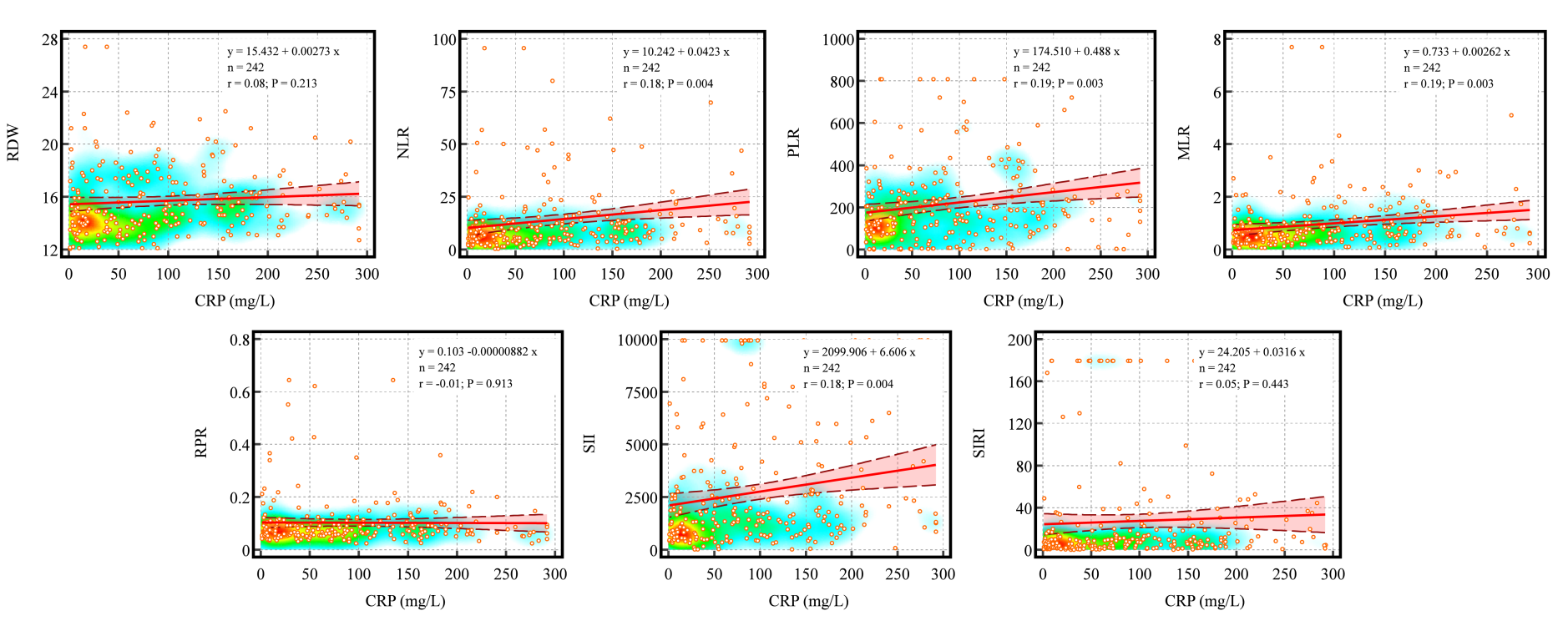


**Supplementary Figure 1.** Analysis of the Correlation Between Inflammatory Markers and CRP. Abbreviations: RDW, red blood cell distribution width; NLR, neutrophil to lymphocyte ratio; PLR, platelet to lymphocyte ratio; MLR, monocyte to lymphocyte ratio; RPR, red blood cell volume distribution width to platelet ratio; SII, systemic immune inflammation index; SIRI, systemic inflammatory response index, CRP, C-reaction protein.
